# Subcellular Localization of Nicotinic Acid Utilization Pathway Proteins in *Aspergillus nidulans* and a Putative HxnX-Dependent Piggyback Import of HxnW

**DOI:** 10.3390/jof12100785

**Published:** 2026-10-20

**Authors:** Suren Nemuuzaya, Judit Ámon, Gabriella Varga, Eszter Bokor, Kitti Vecsernyés-Nagy, Zsuzsanna Hamari

**Affiliations:** 1 Department of Biotechnology and Microbiology, Faculty of Science and Informatics, University of Szeged, 6726 Szeged, Hungary; nemuuzayasuren@gmail.com (S.N.); amon.judit@gmail.com (J.Á.); kaszagabi64@gmail.com (G.V.); b.tikke@gmail.com (E.B.); kittivecsernyesnagy@gmail.com (K.V.-N.); 2 Centre of Excellence for Interdisciplinary Research, Development and Innovation (IKIKK), Competence Centre for Molecular Biology, Bionics and Biotechnology, University of Szeged, 6726 Szeged, Hungary

**Keywords:** nicotinic acid catabolism, peroxisome, peroxisomal targeting signal (PTS1), bimolecular fluorescence complementation (BiFC), metabolic compartmentalization

## Abstract

A recently discovered eukaryotic nicotinic acid catabolic pathway involves the concerted action of eight Hxn enzymes including HxnX, a peroxisomal enzyme carrying a canonical PTS1 motif (SRL) at its C-terminus. None of the seven remaining pathway enzymes contains an obvious peroxisomal targeting signal (PTS). However, the nicotinic acid transporters HxnP and HxnZ, and the 2,5-dihydroxypyridine hydroxylase HxnV, carry non-canonical PTS1 motifs originally described in plants. N-terminal Gfp fusions localized HxnP and HxnZ to the plasma membrane and septa, whereas Gfp-HxnV showed diffuse cytoplasmic localization, with no detectable peroxisomal targeting. The *hxnX* and *hxnW* genes, which encode HxnX and the 5,6-dihydroxypiperidine-2-one ketoreductase HxnW, respectively, are divergently transcribed from a shared intergenic region, suggesting coordinated regulation of their expression and raising the possibility of a functional association between the two proteins. Consistent with this possibility, bimolecular fluorescence complementation (BiFC) provided evidence for an interaction between HxnX and HxnW. However, neither the BiFC fusion proteins nor the Gfp-HxnW fusion complemented the nicotinic acid utilization defects of the *hxnXΔ hxnWΔ* and *hxnWΔ* strains, respectively, and neither showed peroxisomal localization. These findings indicate that fusion of Gfp or split-Yfp fragments interferes with HxnW function, potentially by affecting protein folding, catalytic activity, protein–protein interaction, or intracellular targeting. Thus, while the observed HxnX–HxnW interaction is consistent with a possible piggyback-mediated import mechanism, the peroxisomal import of HxnW remains to be demonstrated experimentally.

## 1. Introduction

A novel eukaryotic primary metabolic pathway enabling *Aspergillus nidulans* to utilize nicotinic acid as the sole nitrogen source was recently discovered [1,2,3]. The pathway comprises two transporters (HxnP and HxnZ), eight enzymes (HxnN, HxnM, HxnS, HxnT, HxnY, HxnX, HxnV and HxnW) and the pathway-specific transcription factor HxnR. Among these proteins, only the 6-hydroxynicotinic acid monooxygenase HxnX carries a canonical PTS1 motif (SRL) and has been experimentally shown to localize to peroxisomes, where it catalyses the conversion of 6-hydroxynicotinic acid to 2,5-dihydroxypyridine [3]. Unexpectedly, the nicotinic acid utilization pathway-specific transporters HxnP and HxnZ, as well as the 2,5-dihydroxypyridine hydroxylase HxnV contain C-terminal sequences matching non-canonical PTS1 motifs originally described in plants [4] and subsequently shown to have predictive value for peroxisomal targeting in fungi [5]. The presence of PTS1-like sequences in HxnP and HxnZ is particularly intriguing because these proteins are predicted membrane transporters and would therefore be expected to localize primarily to the plasma membrane. We therefore investigated whether these C-terminal motifs are associated with detectable peroxisomal localization, which could indicate an additional intracellular pool of the transporters. In addition, *hxnX* and *hxnW*, encoding HxnX and the 5,6-dihydroxypiperidine-2-one ketoreductase HxnW, respectively, are divergently transcribed from a shared intergenic region [2], suggesting coordinated expression and raising the possibility that HxnW enters peroxisomes by piggybacking on HxnX.

Here, we investigated the subcellular localization of HxnP, HxnZ, HxnV and HxnW using N-terminal fusions with green fluorescent protein (Gfp). We show that HxnP and HxnZ localize to the plasma membrane and septa, whereas HxnV remains cytoplasmic. In addition, we obtained evidence for an interaction between HxnW and the peroxisomal enzyme HxnX by using a bimolecular fluorescence complementation (BiFC) assay based on a split-yellow fluorescent protein (Yfp) system optimized for *Aspergillus*. The observed interaction between HxnX and HxnW is consistent with a possible piggyback-mediated mechanism for HxnW import into peroxisomes.

## 2. Materials and Methods

### 2.1. Strains and Media

*A. nidulans* strains used in this study are listed in Table 1. Standard *Aspergillus* genetic markers and minimal medium (MM) are described at the http://www.fgsc.net/Aspergillus/gene_list/ (accessed on 1 November 2008). Media were supplemented with vitamins according to the requirements of each auxotrophic strain, including 40 µg/L pantothenic acid; 40 µg/L para-aminobenzoic acid; 25 µg/L pyridoxine; 50 µg/L riboflavin and 0.1 µg/L biotin. Uracil and uridine were added at 0.1% (*w*/*v*) each, where required for auxotrophic strains. The carbon and nitrogen sources used in the experiments were as follows: 1% (*w*/*v*) glucose as a carbon source; 10 mM sodium nitrate, 10 mM nicotinic acid, or 10 mM 6-hydroxynicotinic acid as nitrogen sources; and 10 mM threonine as the sole carbon and nitrogen source. Nicotinic acid and 6-hydroxynicotinic acid were added as a 1:100 dilution of a 1 M stock solution prepared in 1 M sodium hydroxide. For induction of constructs under the control of the ethanol-inducible *alcA* promoter (P*_alcA_*), ethanol was added to the medium at a final concentration of 1% (*v*/*v*). Strains were grown at 25 °C for microscopy and at 37 °C for complementation tests.

Explanation of mutant alleles, which are not described in the text: *veA1* mutation in *velA* results in profuse conidiation regardless of the presence or absence of light [13]; *nkuA* deletion (nkuAΔ) decreases the frequency of nonhomologous recombinations [9]; *hxnR^c^7* constitutive mutation in HxnR, the transcription factor of the nicotinic acid utilization pathway genes, which results in the activation of the pathway genes when the physiological inducer metabolite, is absent [1]. Other gene symbols refer to auxotrophies: *biA1*, biotin; *pabaA1*, p-aminobenzoic acid; *pantoB100*, pantothenic acid; *pyroA4*, pyridoxine; *riboB2*, riboflavin; *argB2*, arginine; *pyrG89*, uracil and uridine. The *pyr4* gene of *Neurospora crassa* is homologous to the *pyrG* gene of *A. nidulans* and was used to complement the *pyrG89* mutation.

### 2.2. Construction of Gfp-HxnP, Gfp-HxnZ, Gfp-HxnV and Gfp-HxnW Expression Strains

The *gfp-hxnP*, *gfp-hxnZ*, *gfp-hxnV*, and *gfp-hxnW* expression constructs were generated as bipartite *gfp-hxn* fusion cassettes using the Double-Joint PCR (DJ-PCR) method [7]. For all fusions, the *gfp* coding sequence was amplified from plasmid pAN-HZS-1 [14] using primers “1pGpd int frw” (5′-cagtatattcatcttcccatccaagaac-3′) and “10GFP linker rev” (5′-atcaagatcgactgtatcaataagcttgtacagctcgtccatgccgtg-3′). The reverse primer annealed to the 3′ end of *gfp* (excluding the stop codon) and carried a 24-bp 5′ extension (italicized) encoding an eight-amino-acid linker (LIDTVDLD). Prior to amplification of *hxnP* for construction of the *gfp-hxnP* fusion, an internal NcoI restriction site in *hxnP* was silently mutated from CCATGG to CCGTGG. An 1852-bp *hxnP* coding sequence was then amplified using “hxnP kim linker frw” (5′-*gtacaag*cttattgatacagtcgatcttgatatgggcgccactgccaccgat-3′) and “hxnP do R3” (5′-ggagtctatactgcaggattagctg-3′) primers. The forward primer contained a chimeric 5′ extension consisting of a 7-nt sequence corresponding to the region immediately upstream of the *gfp* stop codon (italicized) and the 24-nt linker sequence (underlined). This chimeric extension enabled fusion of the *hxnP* PCR product to the linker-tagged *gfp* fragment during the assembly PCR. The assembly PCR was performed using the nested primer pair “Gfp NcoI frw” (5′-ttttttttccatggtgagcaagggcgaggagctgttc-3′) and “hxnP BamHI rev” (5′-ttttttttggatccctacagaaaataccgccactctggac-3′). The resulting *gfp-hxnP* PCR product was cloned into NcoI-BamHI-digested pAN-HZS-1 [14]. The resulting plasmid, pAN-HZS-41, expressed the *gfp-hxnP* fusion under the control of the constitutive *gpdA* promoter and carried the *pantoB^+^* as a selectable marker for transformation.

The *gfp-hxnZ*, *gfp-hxnV* and *gfp-hxnW* fusion sequences were constructed using the same strategy as described above for *gfp-hxnP*. The *hxnZ*, *hxnV* and *hxnW* genes were amplified using the primer pairs “hxnZ kim linker frw” (5′-*gtacaag*cttattgatacagtcgatcttgatatggatatatcataccctgtcatcaatgctg-3′)—“hxnZ term rev” (5′-gtaagcagcacttccatcgcaagg-3′), “hxnV kim linker frw” (5′-*gtacaag*cttattgatacagtcgatcttgatatggctcgcagcgcagatcac-3′)—“hxnV do307 R” (5′-gatgatcaatgcatacagcactaccac-3′) and “hxnW kim linker frw” (5′-*gtacaag*cttattgatacagtcgatcttgatatgtccaaggtgagactcttccttttc-3′)—“hxnW do R” (5′-actcctccacccaccgtcttg-3′), respectively. Assembly PCRs of the *gfp* and *hxn* fragments were performed using nested forward primer “Gfp NcoI frw” together with the nested reverse primers “hxnZ BamHI rev” (5′-ttttttttggatccctagatagcagcatgcccatgagtctc-3′), “hxnV N NotI rev” (5′-ttttttttgcggccgctcacaagaagaatccaaaatactcctcc-3′) and “hxnW NotI rev” (5′-ttttttttgcggccgcctatgagattctagtcactcccccatc-3′), respectively. The resulting *gfp-hxnZ* (2623-bp) PCR product was cloned into NcoI-BamHI-digested pAN-HZS-1 to generate pAN-HZS-42. The *gfp-hxnV* (2941 bp) and *gfp-hxnW* (1602-bp) PCR products were cloned into NcoI-NotI-digested pAN-HZS-1 to generate pAN-HZS-43 and pAN-HZS-21, respectively.

Plasmid pAN-HZS-21, carrying *gfp-hxnW* and the *pantoB^+^* selectable marker, was transformed into a *hxnWΔ* recipient strain HZS.942. Plasmids pAN-HZS-41, and pAN-HZS-43, carrying *gfp*-*hxnP* and *gfp*-*hxnV*, respectively, together with the *pantoB^+^* selectable marker were transformed into recipient strain HZS.98. Similarly, plasmid pAN-HZS-42, carrying *gfp-hxnZ* and the *pabaA^+^* selectable marker, was transformed into recipient strain HZS.1069.

Transformants were selected for pantothenic acid or *para*-aminobenzoic acid prototrophy. Isolates carrying the respective transgenes were obtained. Transgene copy numbers were determined by qPCR according to the method described by Herrera et al. [15], using the following gene-specific primer pairs: “hxnW ReTi frw” 5′-ggtatggcggtttatccctg-3′ and “hxnW ReTi rev” 5′-cctctcaggattccttgaaagac-3′; “hxnP Reti frw” 5′-tgtacttctttgactgcatgg-3′ and “hxnP Reti rev” 5′-gagtattcgttgcccttgag-3′; “hxnZ Reti frw” 5′-cgctgtatttcaactttctccc-3′ and “hxnZ Reti rev” 5′-cagtagttgcggtaggtcag-3′; and “hxnV Reti frw” 5′-gacccgtatattctcctcgtg-3′ and “hxnV Reti rev” 5′-gaaatgaactattccgcgtaacag-3′. The *actA* gene (encoding γ-actin) was used as an internal control and amplified using primers “actin ReTi frw2” (5′-accatgtaccctggtatctc-3′) and “actin ReTi rev2” (5′-ggaggagcaatgatcttgac-3′). Transformants carrying single or multiple copies of the respective expression cassette were identified, and those with the lowest available copy number were selected for further analysis, preferentially single-copy transformants whenever available. The single-copy strains HZS.917 (for *gfp-hxnW*) and HZS.1072 (for *gfp-hxnV*), the two-copy strain HZS.1070 (for *gfp-hxnP*) and four-copy strain HZS.1071 (for *gfp-hxnZ*) were selected for further analysis.

### 2.3. Construction of an mCherry-SKL (Peroxisome-Targeted mCherry) Expression Vector

The coding sequence of the red fluorescent protein mCherry contains an internal NcoI restriction site that interferes with NcoI-BamHI cloning of the mCherry coding sequence into the pAN-HZS-1 vector [14]. Therefore, prior to cloning, the internal NcoI restriction sequence was silently mutated from CCATGG to CCATAG using the DJ-PCR method [7]. To generate the modified fragment, regions upstream and downstream of the internal NcoI site, with a 17-bp overlap, were amplified using the primer pairs “hhoA F” (5′-cgtcaccacctcttcgcgctatg-3′) and “mCh int errncoI R” (5′-cccatagtcttcttctgcattacg-3′); and “mCh int errncoI F1” (5′-cagaagaagactatgggctgggaggcctcctc-3′) and “Afu pyrG int R” (5′-attgatagtgtcgacgctgaaatcgg-3′), respectively. Genomic DNA from strain LO1944, which carries an integrated “*hhoA*-*mCherry*-*Afu pyrG*” cassette [6], was used as the template. The nucleotide substitution within the NcoI recognition sequence is underlined in the primer sequence. The two PCR fragments were assembled into a single fragment by nested PCR using the primers “mCh NcoI F” (5′-ttttttttccatggtgagcaagggcgag-3′) and “mCh BamHI rev” (5′-ttttttttggatccttataacttagacttgtacagctcgtccatgccg-3′), which introduced NcoI and BamHI restriction sites, respectively. The reverse primer annealed to the 3′ end of the mCherry coding sequence and incorporated a sequence encoding the SKL peroxisomal targeting signal immediately upstream of the stop codon. The assembled mCherry-SKL coding sequence was cloned into NcoI-BamHI-digested pAN-HZS-1. The resulting plasmid, pAN-HZS-52, expresses mCherry-SKL under the control of the constitutive *gpdA* promoter and carries the *pantoB^+^* as a selectable marker. This plasmid was used for transformation of strain HZS.971, yielding strain HZS.1073.

To replace the *pantoB^+^* selectable marker with the *Aspergillus fumigatus pabaA^+^* marker, the *A. fumigatus* (*Afu*) *pabaA^+^* cassette was amplified from genomic DNA of *A. fumigatus* strain 293 using the primer pair “Afu paba NdeI fw” (5′-ttttttcatatgcgagtgcttccatggtcgttgagag-3′) and “Afu paba NdeI rev” (5′-ttttttcatatgcctgcagatagactagagctgccg-3′). The resulting PCR product was cloned into the NdeI restriction site of plasmid pAN-HZS-52, yielding plasmid pAN-HZS-53. This plasmid was used to introduce the mCherry-SKL peroxisomal marker into strain HZS.98, yielding strain HZS.1069. This strain was subsequently transformed with the *gfp-hxnZ*-expression vector pAN-HZS-42 to obtain strain HZS.1071, allowing the localization of Gfp-HxnZ to be compared directly with that of peroxisomes.

### 2.4. Bimolecular Fluorescence Complementation (BiFC) Analyses

For BiFC analyses, the full-length *hxnW* and *hxnX* genes were amplified from genomic DNA using primers containing *Asc*I and *Pac*I restriction sites at their 5′ ends. *hxnW* was amplified using primers “hxnW AscI frw” 5′-ttttt*ggcgcgcc*catgtccaaggtgagactcttcc-3′ and “hxnW PacI rev” 5′-ttttt*ttaattaa*caaattacctatgagattctagtcactcc-3′, whereas *hxnX* was amplified using primers “hxnX AscI frw” 5′-ttttt*ggcgcgcc*catgccagttgcagagaaacaatttcg-3′ and “hxnX PacI rev” 5′-ttttt*ttaattaa*tcataaccgcgatgctacctgttc-3′. Italicized sequences indicate the restriction sites. Following restriction digestion, the *hxnW* PCR product was cloned into pDV7 [16], yielding plasmid pDV7-W, which carries a P*_alcA_*::*yfp*_N_::*hxnW* fusion. Similarly, the *hxnX* PCR product was cloned into pDV8 [16], yielding plasmid pDV8-X, which carries a P*_alcA_*::*yfp*_C_::*hxnX* fusion. As negative controls for non-specific fluorescence complementation, recipient strain HZS.963 was co-transformed with pDV7-W and empty pDV8 vector, or with the empty pDV7 vector and pDV8-X. No fluorescence was detected in either control. For BiFC analysis of the HxnW-HxnX interaction, HZS.963 was co-transformed with pDV7-W and pDV8-X. The transformants were selected for uracil and uridine prototrophy, and integration of the constructs was confirmed by qPCR. The *hxnW*-specific primers “hxnW ReTi frw” and “hxnW ReTi rev” were used as described above. The *hxnX-*specific primers were “hxnX ReTi frw” 5′-cttgtatcatctccacgacgg-3′ and “hxnX ReTi rev” 5′-ggctaaacactctccctctg-3′ and the *actA* (encoding γ-actin) specific primers “actin ReTi frw2” and “actin ReTi rev2” were used as described above. Transformant HZS.971, carrying two copies each of the P*_alcA_*::*yfp*_N_::*hxnW*-*pyr4^+^* and P*_alcA_*::*yfp*_C_::*hxnX*-*pyr4^+^* constructs, was selected for further analysis.

### 2.5. Microscopy

Conidiospores of the strains analysed were germinated on coverslips submerged in MM under the conditions specified in the respective figure legends at 25 °C. Young hyphae were examined by fluorescence microscopy using a Zeiss Axio Observer.Z1/7 microscope equipped with an HXP 120 V mercury short-arc lamp (Zeiss, Jena, Germany). Zeiss filter set 43 HE was used for mCherry fluorescence detection (excitation: 538–562 nm; beam splitter: 570 nm; emission: 570–640 nm) and 38 HE was used for Gfp and Yfp fluorescence detection (excitation: 450–490 nm; beam splitter: 495 nm; emission: 500–550 nm).

## 3. Results

### 3.1. Localization of HxnP, HxnZ and HxnV Carrying Non-Canonical PTS1 Motifs

**Figure 1:**
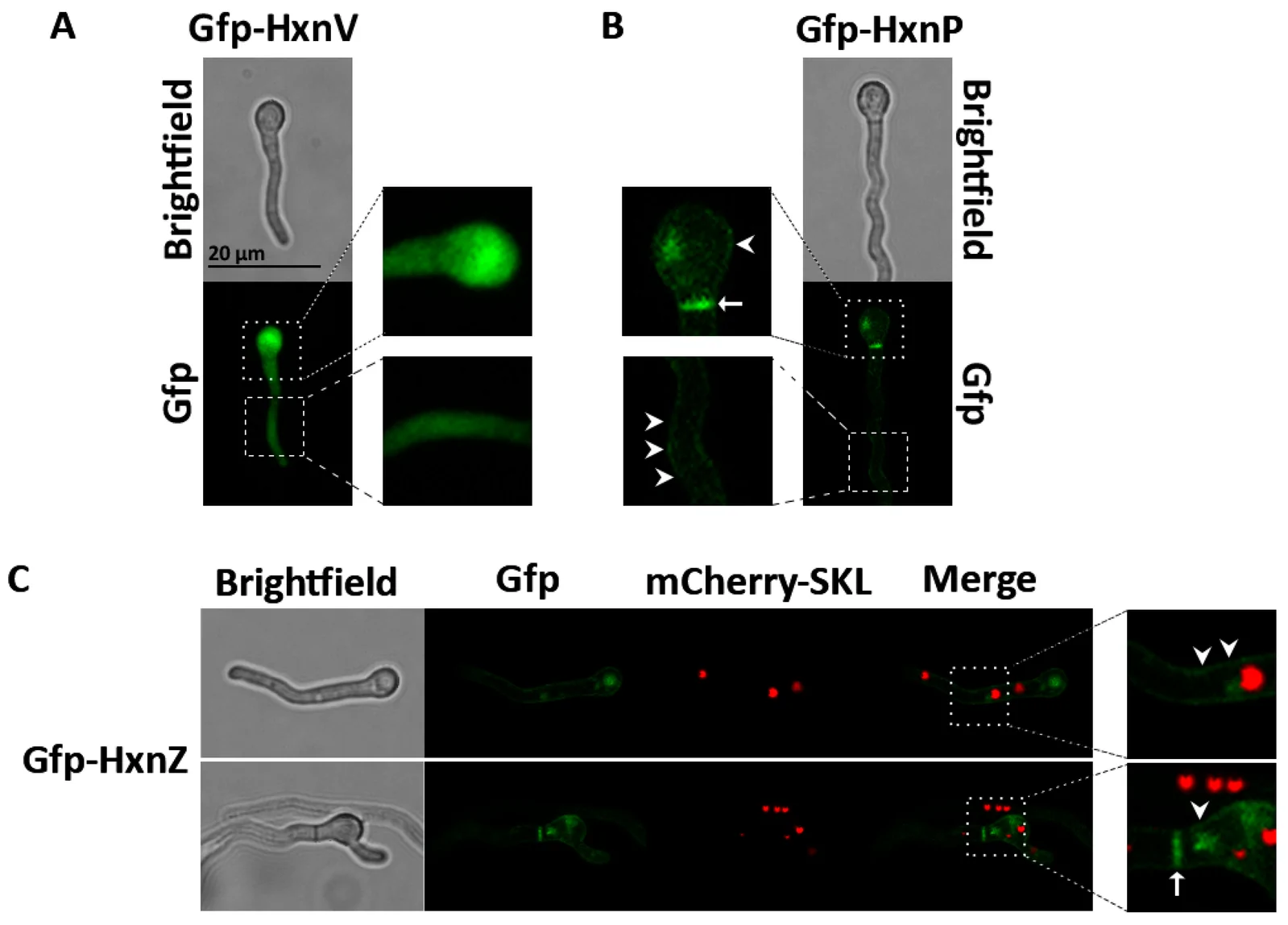
Subcellular localization of HxnV, HxnP, and HxnZ in *A. nidulans*.

Although several subcellular localization prediction tools (Deeploc 2.1, PredPlant PTS1, WoLF PSORT, and CELLO v.2.5) did not predict peroxisomal localization for any of the Hxn proteins except HxnX, three proteins, HxnP, HxnZ and HxnV contain C-terminal sequences matching non-canonical plant-type PTS1 motifs [4] (YFL, AAI, and FFL, respectively). Because related PTS1 consensus sequences have recently been shown to have predictive value in fungi [5], we tested whether these motifs were associated with detectable peroxisomal localization of HxnP, HxnZ and HxnV. To this end, N-terminal Gfp fusions of HxnP, HxnZ, and HxnV were expressed in *A. nidulans*. Gfp-HxnZ and Gfp-HxnP localized to the plasma membrane and septa, whereas Gfp-HxnV displayed diffuse cytoplasmic localization (Figure 1). No peroxisomal localization was detected for any of the three proteins. In particular, the punctate Gfp-HxnZ fluorescence showed no apparent co-localization with the mCherry-SKL peroxisomal marker (Figure 1C). These experimental observations were consistent with the predictions of Deeploc 2.1, PredPlant PTS1, WoLF PSORT, and CELLO v.2.5.

Representative microscopy images of *A. nidulans* strains expressing N-terminally Gfp-tagged HxnV (HZS.1072), HxnP (HZS.1070) or HxnZ (HZS.1071) under the control of the constitutive *gpdA* promoter. (A) Gfp fluorescence (green) and corresponding brightfield images of Gfp-HxnV, showing diffuse cytoplasmic fluorescence. (B) Gfp fluorescence and corresponding brightfield images of Gfp-HxnP, showing fluorescence associated with the plasma membrane and septa. (C) Gfp fluorescence and corresponding brightfield images of Gfp-HxnZ. Gfp-HxnZ showed fluorescence associated with the plasma membrane and septa, and its punctate fluorescence showed no apparent co-localization with the mCherry-SKL peroxisomal marker (red). Strains were grown overnight at 25 °C in minimal medium containing 1% glucose as the carbon source and 10 mM sodium nitrate as the nitrogen source. White arrowheads indicate plasma membrane-associated fluorescence, whereas white arrows indicate septal fluorescence. Enlarged views of the indicated regions are provided in each panel. Scale bar indicates 20 µm.

### 3.2. HxnW Shows Evidence of Interaction with the Canonical PTS1-Containing Protein HxnX

HxnW contains neither a PTS1 nor a PTS2 targeting signal. However, *hxnW* shares a common intergenic region with the peroxisomal *hxnX* gene, and the two genes are divergently transcribed [2,3]. This genomic organization suggests coordinated expression and prompted us to investigate whether HxnW interacts with HxnX.

**Figure 2:**
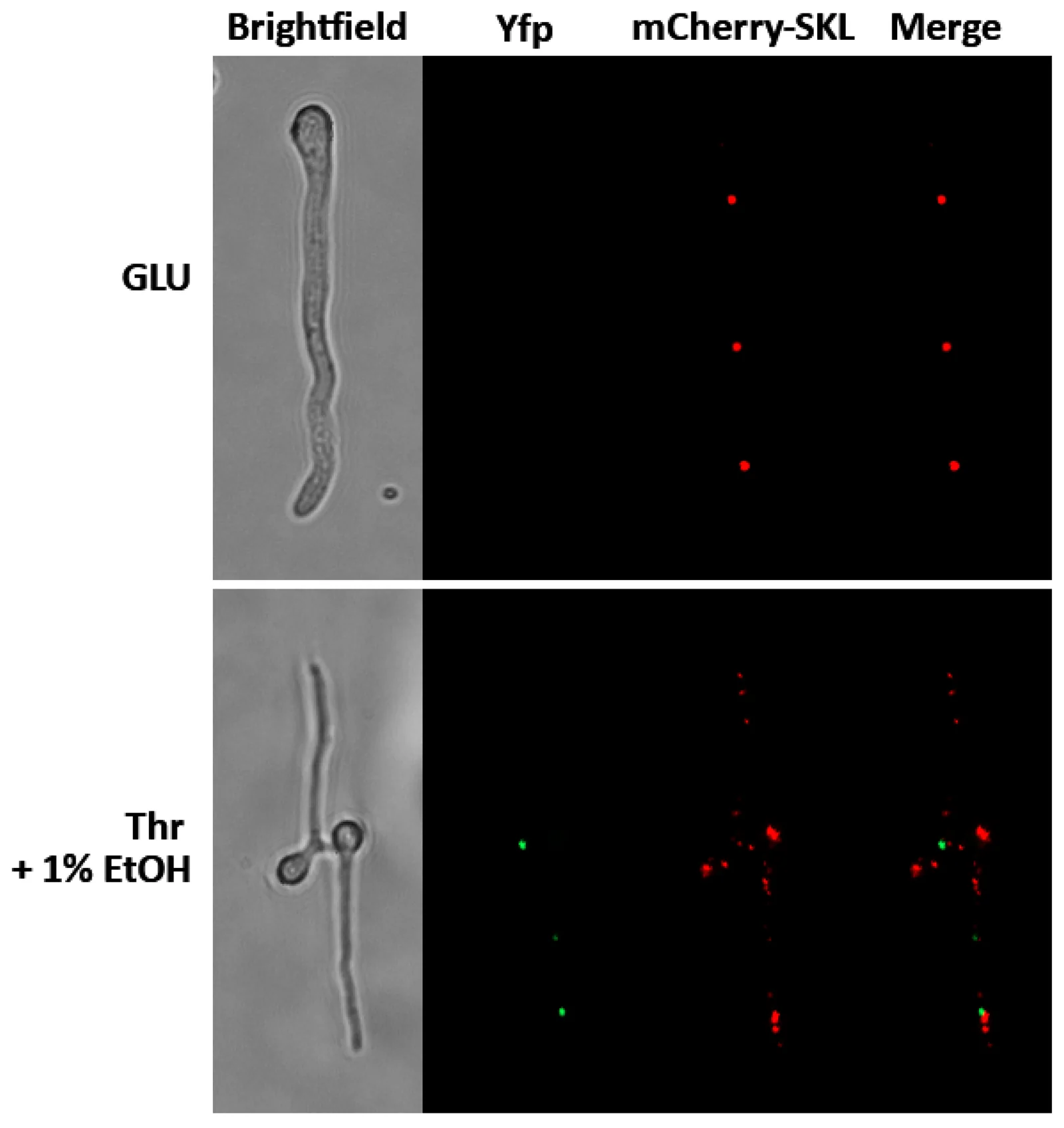
Analysis of the interaction between HxnW and HxnX by bimolecular fluorescence complementation.

To test this hypothesis, we performed BiFC by fusing the N-terminal half of Yfp to HxnW and the C-terminal half of Yfp to HxnX. The fusion constructs were co-expressed in an *hxnXΔ hxnWΔ* double-deletion recipient strain (HZS.963). Both fusion genes were placed under the control of the ethanol-inducible, glucose-repressible *alcA* promoter (P*_alcA_*) [16]. Fluorescence was examined in the transformant strain HZS.971 after 2 h of induction in threonine medium supplemented with 1% ethanol. Under inducing conditions, the Yfp fluorescence signal appeared as distinct puncta, whereas no fluorescence was detected in glucose-containing medium, consistent with the repression of the *alcA* promoter. To determine whether these fluorescent puncta corresponded to peroxisomes, the BiFC strain HZS.971 was transformed with the mCherry-SKL peroxisomal marker plasmid (pAN-HZS-52), generating strain HZS.1073. No apparent co-localization between the BiFC signal and the peroxisomal marker was observed in HZS.1073 (Figure 2). No fluorescence was detected in either negative-control combination, in which HZS.963 was co-transformed with pDV7-W and empty pDV8 or with empty pDV7 and pDV8-X, indicating that the BiFC signal required the presence of both HxnW- and HxnX-fused Yfp fragments.

BiFC analysis was performed in strain HZS.1073, which carries the *hxnXΔ hxnWΔ* double deletion and expresses the mCherry-SKL peroxisomal marker (red) and split-Yfp fragments (functional Yfp is green) fused to HxnW and HxnX. Expression of the fusion genes was controlled by the ethanol-inducible, glucose-repressible *alcA* promoter (*P_alcA_*). Cells were grown overnight at 25 °C on 1% glucose (GLU, repressing condition) or on 10 mM threonine followed by induction with 1% ethanol for 2 h (Thr + 1% EtOH). Scale bar indicates 20 µm.

The BiFC strain was unable to utilize nicotinic acid or 6-hydroxynicotinic acid as the sole nitrogen source, indicating that the split-Yfp fusion proteins did not complement the *hxnXΔ hxnWΔ* mutations. A Gfp-HxnW fusion protein expressed in an *hxnWΔ* background (HZS.917) also failed to complement the *hxnWΔ* mutation. Thus, although the BiFC signal provides evidence for an interaction between HxnX and HxnW, the fusion proteins were non-functional and failed to show the expected peroxisomal localization. These findings indicate that the fluorescent protein fusions interfere with the normal function of HxnW and/or HxnX, potentially by affecting protein folding, catalytic activity, protein–protein interaction, or intracellular targeting.

## 4. Discussion

The presence of non-canonical PTS1-like sequences at the C-termini of HxnP and HxnZ raised the possibility that, in addition to their expected localization at the plasma membrane, a fraction of these transporters might be targeted to peroxisomes. Such an alternative localization could potentially contribute to the intracellular distribution of the transporters, for example by sequestering a fraction of these proteins away from the plasma membrane. Our localization experiments, however, provided no evidence for such a peroxisomal population. Both Gfp-HxnP and Gfp-HxnZ localized to the plasma membrane and septa, consistent with their proposed roles as membrane transporters. Nevertheless, our experiments cannot exclude condition-dependent targeting or a minor peroxisome-associated population below the detection limit of fluorescence microscopy. It also remains possible that these C-terminal motifs have functions unrelated to peroxisomal targeting. Determining whether the motifs themselves influence transporter localization, stability or turnover would require their targeted mutation and comparison of the localization and abundance of the resulting proteins with those of the wild-type transporters.

The case of HxnV is distinct from that of the two transporters because HxnV is a soluble enzyme and therefore represents a more plausible candidate for PTS1-mediated import into the peroxisomal matrix. HxnV carries a C-terminal FFL sequence matching a non-canonical PTS1 motif, raising the possibility that the conversion of 2,5-dihydroxypyridine to 2,3,6-trihydroxypyridine might constitute an additional peroxisomal step of the pathway. However, Gfp-HxnV showed diffuse cytoplasmic localization with no detectable enrichment in peroxisomes. Our results therefore provide no experimental support for peroxisomal localization of HxnV under the conditions examined and suggest that the HxnV-catalyzed reaction predominantly occurs in the cytoplasm. As with HxnP and HxnZ, condition-dependent or low-level peroxisomal localization cannot be excluded, and the functional significance, if any, of the C-terminal FFL sequence remains unresolved.

Piggyback-mediated import into peroxisomes has been reported for only a limited number of proteins. Examples include the co-import of Eci1p (delta3,delta2-enoyl-CoA isomerase) with its paralogue Dci1p (delta(3,5)-delta(2,4)-dienoyl-CoA isomerase) in *Saccharomyces cerevisiae* [17,18], the co-import of Pnc1p (nicotinamidase) with the PTS2-containing Gpd1p (glycerol-3-phosphate dehydrogenase) in *S. cerevisiae* [19,20,21], the import of the heteropentameric acyl-CoA oxidase complex (composed of five isoforms of acyl-CoA oxidase, of which only two, Aox3p and Aox5p, carry PTS signals) from *Yarrowia lipolytica* [22,23] and the co-import of mammalian SOD1 (Cu/Zn superoxide dismutase 1) with its copper chaperone CCS, which carries a non-canonical PTS1 signal (AHL) [24].

Although our fluorescence microscopy experiments did not provide direct evidence for peroxisomal import of HxnW, several observations are consistent with this possibility. Neither Gfp-HxnW nor the BiFC fusion proteins complemented the nicotinic acid utilisation-deficient phenotypes of the *hxnWΔ* and *hxnXΔ hxnWΔ* mutants, respectively. Furthermore, neither construct showed detectable peroxisomal localization. Thus, the fluorescent protein fusions appear to interfere with the normal function of HxnW and/or HxnX, although the underlying mechanism cannot be determined from the present experiments. The tags may affect protein folding or catalytic activity, interfere with HxnX–HxnW interaction or its appropriate stoichiometry, or alter intracellular targeting. Consequently, the absence of detectable peroxisomal localization of these fusion proteins cannot be taken as evidence that native HxnW is excluded from peroxisomes. Conversely, however, our experiments provide no direct evidence that native HxnW is peroxisomal. The present data therefore cannot distinguish between a scenario in which fluorescent tagging interferes with the normal localization of HxnW and one in which native HxnW is not targeted to peroxisomes. Resolving this ambiguity will require localization of a functional HxnW protein and testing whether any observed peroxisomal targeting depends on HxnX.

BiFC analysis provided evidence for an interaction between HxnX and HxnW. Although this observation does not demonstrate piggyback-mediated import, interaction between the two proteins is a prerequisite for such a mechanism. Importantly, however, the BiFC signal did not co-localize with the mCherry-SKL marker and therefore provides no evidence that the observed HxnX–HxnW association occurs in peroxisomes.

Nevertheless, the presence of a canonical PTS1 in HxnX, the absence of an identifiable peroxisomal targeting signal in HxnW, and the observed association between the two proteins provide a rationale for a model in which HxnW associates with HxnX in the cytoplasm and is subsequently co-imported into the peroxisomal matrix.

An additional argument for a functional association between the two proteins comes from the genomic organization of the pathway. The *hxnX* and *hxnW* genes share a bidirectional promoter and are divergently transcribed under the control of the pathway-specific transcription factor HxnR [2]. This genomic arrangement is highly conserved across several genera of Eurotiomycetes, including *Aspergillus, Penicillium,* and *Talaromyces*, suggesting that the coordinated expression of *hxnX* and *hxnW* may be functionally important, as documented in the comparative analysis presented in the Supplementary Material of our previous study [2]. Coordinated expression of the two genes would also be compatible with cytoplasmic association of HxnX and HxnW before their proposed translocation into peroxisomes. Such a mechanism could enable the coordinated delivery of the two interacting enzymes into peroxisomes, with HxnW relying on the targeting signal carried by HxnX rather than requiring its own identifiable PTS. Such coordinated import may help maintain appropriate levels of both enzymes within the organelle and facilitate efficient progression of the pathway.

Moreover, piggyback-mediated import could provide a simple mechanism to ensure that HxnW is imported only when HxnX is present. This could prevent accumulation of HxnW in peroxisomes when HxnX, which functions further upstream in the pathway, is absent. Additionally, because accumulation of the substrates of both enzymes (6-hydroxynicotinic acid for HxnX and 5,6-dihydroxypiperidine-2-one for HxnW) is toxic to the cell [3], coordinated import of the two enzymes may also minimize the accumulation of toxic intermediates and thereby improve the efficiency of nicotinic acid catabolism. Direct demonstration of HxnW piggyback import, however, will require localization of functional HxnW and evidence that its peroxisomal targeting depends on HxnX.

Several limitations should be considered when interpreting the proposed HxnX-dependent piggyback import of HxnW. Neither Gfp-HxnW nor the split-Yfp fusion proteins were functional, and therefore their observed localization may not reflect that of native HxnW. Furthermore, although BiFC provided evidence for an HxnX–HxnW interaction, the BiFC signal did not co-localize with the peroxisomal marker and therefore does not demonstrate that the interaction occurs in peroxisomes or results in co-import. The dependence of HxnW localization on HxnX and its PTS1-mediated import pathway was not tested directly.

## Figures and Tables

**Table 1:** List of *A. nidulans* strains used in this work.

Strain	Genotype	Purpose	Reference
LO1944	*hhoA-mCh*-*AfuPyrG riboB2 pyrG89 pyroA4 nkuA∆::argB argB2*	total DNA was used as template for PCR	[6]
CS2902	*pyrG89 riboB2 pyroA4 biA1 veA1*	parental strain in genetic cross to obtain HZS.963	[7]
HZS.98	*pantoB100 pabaA1 veA1*	recipient strain in transformation experiment to obtain Gfp-HxnP, Gfp-HxnV and mCherry-SKL strains	[1]
HZS.145	*veA1*	total DNA was used as template for PCR	[8]
HZS.388	*hxnWΔ::riboB^+^ nkuA* *Δ* *::argB^+^ riboB2 pyroA4 veA1*	parental strain in genetic cross to obtain HZS.942	this work (by transformation of the “uphxnW-riboB^+^-downhxnW” substitution cassette [2] into TN02 A21 [9])
HZS.572	*pantoB100 pabaA1 biA1 veA1* and *in trans* 1 copy of *pDsRed-SKL-argB^+^* plasmid	parental strain in genetic cross to obtain HZS.942	this work (obtained by genetic cross of NA0313 [10] and NA1322 [11])
HZS.751	*hxnXW* *Δ* *::riboB^+^ riboB2 hxnR^c^7 pantoB100 veA1*	parental strain in genetic cross to obtain HZS.963	[2]
HZS.917	*hxnWΔ::riboB^+^ pantoB100 biA1 pabaA1 veA1* and *in trans* 1 copy of P*_gpdA_*-*gfp*-*hxnW-pantoB^+^* plasmid and *in trans* 1 copy of *pDsRed-SKL-argB^+^* plasmid	complementation tests	this work (by transformation of the pAN-HZS-21 into HZS.942)
HZS.942	*hxnWΔ::riboB^+^ pantoB100 biA1 pabaA1 veA1* and *in trans* 1 copy of *pDsRed-SKL-argB^+^* plasmid	recipient strain in transformation experiment to obtain Gfp-HxnW expressing strain	this work (obtained by genetic cross of HZS.572 with HZS.388)
HZS.963	*hxnXΔ-hxnWΔ*::*riboB^+^ hxnR^c^7 pyrG89 riboB2 pyroA4 biA1 pantoB100 veA1*	recipient strain in transformation experiments to obtain P*_alcA_*::*yfp*_N_::*hxnW* and P*_alcA_*::*yfp*_C_::*hxnX* co-expressing strain	this work (obtained by genetic cross of HZS.751 with CS2902)
HZS.971	*hxnXΔ-WΔ::riboB^+^ hxnR^c^7 pyrG89 riboB2 pyroA4 biA1 pantoB100 veA1* and *in trans* 2 copies of P*_alcA_*::*yfp*_N_::*hxnW* plasmid (pDV7-W) and 2 copies of P*_alcA_*::*yfp*_C_::*hxnX* plasmid (pDV8-X)	fluorescent microscopy (BiFC analysis), complementation tests	this work (by transformation of pDV7-W and pDV8-X plasmids into HZS.963)
HZS.1069	*pantoB100 pabaA1 veA1 and mCherry-SKL in trans* (2 ectopically integrated copies of the pAN-HZS-53 vector expressing *mCherry-SKL* under the control of *P_gpdA_* promoter)	recipient strain in transformation experiments to obtain Gfp-HxnZ expressing strain	this work (by transformation of pAN-HZS-53 plasmid into HZS.98)
HZS.1070	*pantoB100 pabaA1 veA1 and gfp-hxnP in trans* (2 ectopically integrated copies of the pAN-HZS-41 vector expressing *gfp-hxnP* under the control of *P_gpdA_* promoter)	fluorescent microscopy	this work (by transformation of pAN-HZS-41 plasmid into HZS.98)
HZS.1071	*pantoB100 pabaA1 veA1* and *mCherry-SKL* (*in trans* 2 copies) and *gfp-hxnZ* (4 ectopically integrated copies of the pAN-HZS-42 vector expressing *gfp-hxnZ* under the control of the *P_gpdA_* promoter)	fluorescent microscopy	this work (by transformation of pAN-HZS-42 plasmid into HZS.1069)
HZS.1072	*pantoB100 pabaA1 veA1* and *gfp-hxnV in trans* (1 ectopically integrated pAN-HZS-43 vector expressing *gfp-hxnV* under the control of the *P_gpdA_* promoter)	fluorescent microscopy	this work (by transformation of pAN-HZS-43 plasmid into HZS.98)
HZS.1073	*hxnXΔ-WΔ::riboB^+^ pyrG89 riboB2 pyroA4 biA1 pantoB100 hxnR^c^7 veA1* *in trans* 2 copies of *P_alcA_::yfp_N_::hxnW-pyr4^+^ (pDV7-W),* 2 copies of *P_alcA_::yfp_C_::hxnX-pyr4^+^ (pDV8-X),* and 1 copy of pAN-HZS-52 vector expressing *mCherry-SKL* under the control of *P_gpdA_* promoter	fluorescent microscopy, complementation tests	this work (by transformation of pAN-HZS-52 plasmid into HZS.971)
*Aspergillus fumigatus* 293	wild type	total DNA was used as template for PCR	[12]

## Data Availability

The original contributions presented in this study are included in the article. Further inquiries can be directed to the corresponding author.
